# Cross-Correlation Algorithm-Based Optimization of Aliasing Signals for Inductive Debris Sensors

**DOI:** 10.3390/s20205949

**Published:** 2020-10-21

**Authors:** Xingjian Wang, Hanyu Sun, Shaoping Wang, Wenhao Huang

**Affiliations:** 1 School of Automation Science and Electrical Engineering, Beihang University, Beijing 100191, China; sun.hy@siat.ac.cn (H.S.); shaopingwang@buaa.edu.cn (S.W.); dsn07@buaa.edu.cn (W.H.); 2 Beijing Advanced Innovation Center for Big Data-based Precision Medicine, Beihang University, Beijing 100191, China; 3 Ningbo Institute of Technology, Beihang University, Ningbo 315800, China; 4 Science and Technology on Aircraft Control Laboratory, Beihang University, Beijing 100191, China; 5 Shenzhen Institutes of Advanced Technology, Chinese Academy of Sciences, Shenzhen 518055, China

**Keywords:** inductive debris sensor, signal aliasing, cross-correlation algorithm, optimization strategy

## Abstract

An inductive debris sensor can monitor a mechanical system’s debris in real time. The measuring accuracy is significantly affected by the signal aliasing issue happening in the monitoring process. In this study, a mathematical model was built to explain two debris particles’ aliasing behavior. Then, a cross-correlation-based method was proposed to deal with this aliasing. Afterwards, taking advantage of the processed signal along with the original signal, an optimization strategy was proposed to make the evaluation of the aliasing debris more accurate than that merely using initial signals. Compared to other methods, the proposed method has fewer limitations in practical applications. The simulation and experimental results also verified the advantage of the proposed method.

## 1. Introduction

Wear and friction are common phenomena in mechanical systems. Researchers have found that the cumulative behaviors of wear and tear in mechanical systems are the primary causes of mechanical failure [1,2]. Debris is generated continuously in the process of friction and wear, with many useful characteristics that can reflect the health condition of a mechanical system [3,4]. For example, the size, concentration and cumulation of debris reflect the rate of wearing and the shape, and the components of the debris reflect the location of wear [5,6]. More previous research [7,8] also indicated that the size and concentration of debris vary in different periods of wear. In normal conditions, the size of wear debris is in the range of 1–20 μm; when abnormal wear begins, the size of the debris particles rises up to 50–100 μm; in the meantime, there is an increase in the debris’ concentration; when the middle and late periods of the lifetime of a machine are reached, a large number of debris particles over the size of 200 μm is generated [8,9]. Various methods have been developed to detect oil debris [10,11,12], among which inductive debris sensors have shown great potential, with the advantages of online detection, a simple structure and being insensitive to oil [13].

**Figure 1:**
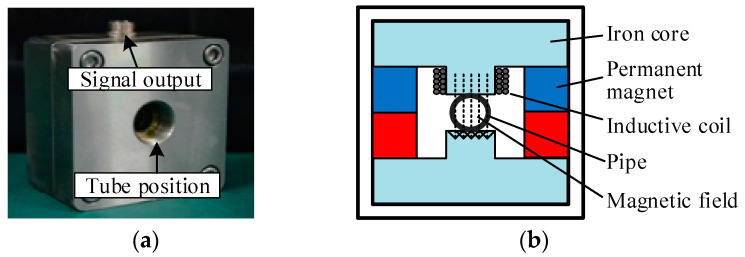
Radial magnetic field-based debris sensor: external (**a**) and internal (**b**) structure.

When oil debris in an oil pipe passes through the sensitive area of an inductive debris sensor, it leaves electromagnetic disturbances in the output signal of the inductive debris sensor [14,15]. It is possible to trace the characteristics of debris by processing the output signal; the number of the output signal waveforms can reflect the number of debris particles, and the peak value of the output signal waveform can reflect the size of the debris. Earlier in 1988, Centers et al. designed an inductive sensor that is able to detect debris over the size of 250 μm [9]; at the same time, an inductive sensor capable of measuring the cumulative size of debris was designed by Chambers et al. [16]. Hong et al. designed a radial magnetic field-based debris sensor that is able to detect ferromagnetic debris over the size of 81 μm in a 12 mm-diameter pipe [17]. Specifically, we utilized this radial magnetic field-based debris sensor in our experiment; the external and internal structure of this sensor is shown in Figure 1. The external structure mainly includes a main body, signal output and tube position for pipe. The internal structure mainly consists of an iron core, permanent magnet and inductive coil [13].

Most of the aforementioned research focused on improving the sensitivity of the inductive debris sensor, in order to detect smaller debris. However, when two debris particles pass through the sensitive area of an inductive sensor within a close distance of each other, their signals alias with each other, which leads to an inaccurate signal. At present, the practical methods for evaluating the number and size of debris particles depend on the number and the peak value of the signal waveform of the debris passing through the inductive sensor [14]. Meanwhile, after the aliasing, the features of the signal waveform, such as the number and peak value, can be influenced. When the peak values are heavily weakened by the aliasing, which can cause a no-detection scenario, this method does not work. Recently, Zhong et al. [18] tried to separate aliasing debris using two debris sensors, but they only focused on the special case of two debris particles located in the same radical direction of the sensor. After this, Li et al. carried out a degenerated unmixing estimation technique to separate aliasing signals [19], but many sophisticated assumptions are made using this method, which may not be applicable in practical systems. The aliasing error can be reduced by studying the debris signal’s aliasing behavior; a further solution to recover the undetected debris waveform can make the results more accurate.

A severe aliasing influence will reduce the peak value to an extent that it becomes below the threshold value or, even worse, below the noise value. Typically, there are three ways to deal with this problem: reducing the threshold value, improving the signal-to-noise ratio (SNR), or improving the affected peak value. Reducing the threshold value will bring in noise signals, which results in a reduction in reliability. Improving the SNR has been frequently studied; Bozchalooi et al. [20] used a two-stage de-noising scheme to eliminate vibration and background noise. Hong et al. [17] presented a hybrid method combining a band pass filter with a correlation algorithm to improve the SNR.

In Hong‘s article [17], he used a correlation algorithm to eliminate random noise and enlarge a single debris signal’s peak value. By operating a correlation algorithm, the similarity (which is the debris’ waveform) between two series is enlarged as a larger peak value. While an aliasing signal can be seen as the mixing of several single debris signals, each single debris signal’s peak value is supposed to be enlarged, respectively, when correlation computing is operated. Inspired by this, we propose operating the cross-correlation of the aliasing signal using a standard debris signal. A mathematical model-based analysis was first performed to explain the aliasing phenomenon as well as the effective range for our proposed method. Afterwards, an optimization strategy was taken to perform a better evaluation of the affected signal’s peak value. In the simulation experiment, the effectiveness of our proposed method in dealing with two debris particles’ aliasing issue was tested; factors such as the influence of noise were also considered in the simulation. At last, a real wax block experiment was performed to verify the efficiency of our method in practice. In both experiments, by using our proposed method, we expected to perform a more accurate evaluation of the affected debris’ peak value than that using the initial unprocessed signal. While there is no need for extra sensors when implementing or calculating the resources for sophisticated algorithms, the verification of our proposed method will solve the debris aliasing issue of the inductive debris sensor in a more practical way.

The rest of the paper is organized as follows: In Section 2, the aliasing issue is analyzed based on a mathematical model. In Section 3, a crossing-correlation algorithm for an aliasing signal is built; a strategy to optimize the aliasing problem is formulated. In Section 4, the performance of the simulation and experiment is presented. In Section 5, some conclusions and remarks are presented.

## 2. Model Analysis of Debris Aliasing Signal

### 2.1. Aliasing Signal Model of Debris Aliasing Behavior

**Figure 2:**
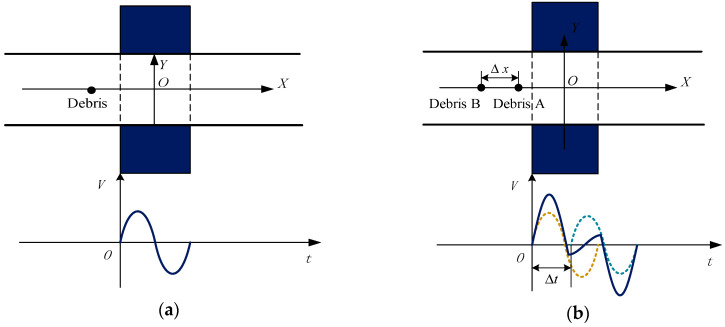
Debris passing the sensor and the signal generated under different situations: (**a**) Single debris particle passing through the sensor; (**b**) Two debris particles passing through the sensor.

Based on our previous research [10,13,21], when a single debris particle passes through the debris sensor, it generates a sinusoid-like signal as shown in Figure 2a. Supposing a piece of debris is passing through the sensor at a constant speed and the time it reaches the sensor is t=0, the output signal of the sensor mixed with noise at a specific frequency and random noise can be defined as

(1) $$V\left(t\right)=\{\begin{array}{l} \lambda \sin\left(\omega t\right)+\lambda_{x}\sin\left(\omega_{n}t\right)+n\left(t\right),\;\left(0\leq t\leq 2\pi /\omega \right)\\ \lambda_{x}\sin\left(\omega_{n}t\right)+n\left(t\right),\begin{array}{cc} \; & \left(t<0\;\mathrm{or}\;2\pi /\omega >t\right) \end{array} \end{array}$$

where λ is the amplitude of the debris signal and is related to the size of the debris [21]; ω is the frequency of the moving debris signal; $\lambda_{x}$ and $\omega_{n}$ are the amplitude and frequency of noise at a specific frequency, respectively; and n(t) is the random noise.

Since the aliasing phenomenon in the practical systems is the superposition of two or multiple two debris particles, it is fundamental to study the aliasing effect of two debris particles.

**Figure 3:**
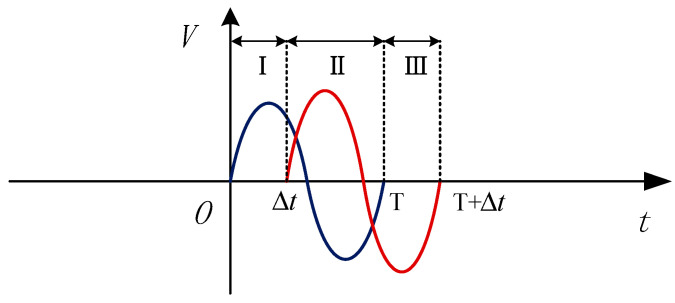
The three segments of the signal model for two aliasing debris.

In an inductive debris sensor, the debris signal generation includes the process of electromagnetic induction and the process of electrical response. Since the superposition theorem is suitable for both processes, the output signal can be simplified as the superposition of the two independent debris signals. Figure 2b shows the process and output signal when Debris A and Debris B pass the debris sensor along the X axis with a distance Δx. It is important to note that all debris moves with the oil and passes through the debris sensor at the same speed. By using the function defined in Equation (1) and ignoring the interference of noise (noise is not the main reason why aliasing occurs, and the SNR was also optimized based on the previous research), we can obtain the signal model for two aliasing debris particles as

(2) $$V\left(t\right)=\{\begin{array}{l} \lambda_{1}\sin\left(\omega t\right),\;t\in \left[0,\Delta t\right)\\ \lambda_{1}\sin(\omega t)+\lambda_{2}\sin[\omega \left(t-\Delta t\right)],\;t\in \left[\Delta t,\;2\pi /\omega \right)\\ \lambda_{2}\sin[\omega \left(t-\Delta t\right)],\;t\in \left[2\pi /\omega ,\;2\pi /\omega +\Delta t\right] \end{array}$$

where we divided the function into three segments (I, II and III), as shown in Figure 3.

Segment I represents the normal signal part of Debris A. Segment II represents the aliasing part of Debris A and B. Segment III represents the normal signal part of Debris B. In Equation (2), $\lambda_{1}$ is the amplitude of the Debris A signal, $\lambda_{2}$ is the amplitude of the Debris B signal, and Δt is the time delay of Debris B. If *T* is set as the time a single debris particle passes through the sensitive area of the sensor, then T=2π/ω.

### 2.2. Analysis of the Aliasing Signal Model

**Figure 4:**
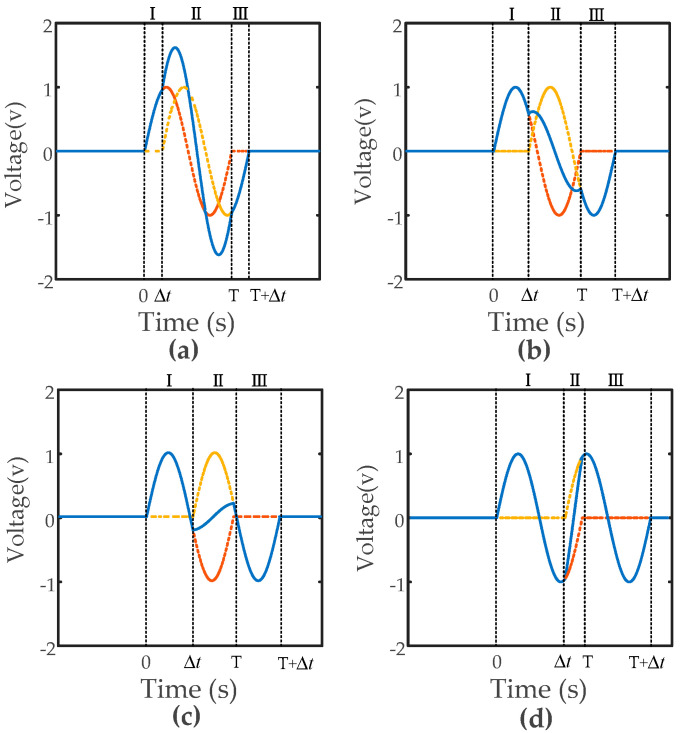
Four typical superposition states: under different situations: (**a**) Δt∈[0, T/4); (**b**) Δt∈[T/4, T/2); (**c**) Δt∈[T/2, 3T/4); (**d**) Δt∈[3T/4, T).

In threshold-based debris detection, the peak values of the detected waveform play an important role in indicating the existence of debris as well as evaluating the debris’ size [5,12], while the peak value is heavily influenced when aliasing happens. In the following step, focusing on figuring out the peak value (here, we only focused on the maximum value) using Equation (2), the behavior of debris aliasing and mechanism behind it is discussed. Considering the number and magnitude of Equation (2)’s maximum value varies with the change in Δt, we divided the superposition results into four different states as shown in Figure 4.

- When Δt∈[0, T/4)

This case as shown in Figure 4a represents the situation in which the distance between two debris particles is very narrow. It can be calculated that $V^{\prime }\left(t\right)>0$ when in Segment I and $V^{\prime }\left(t\right)<0$ when in Segment III, so it is obvious that the peaks are not in these two segments. The peak exists in Segment II.

In Segment II, let $V^{\prime }\left(t\right)=\omega \lambda_{1}\cos\omega t+\omega \lambda_{2}\cos\omega \left(t-\Delta t\right)=0$; two peaks can be obtained. The time index of the maximum is

(3) $$t_{0}=\frac{\pi }{\omega }-\frac{1}{\omega }\arctan\frac{\lambda_{1}+\lambda_{2}\cos\left(\omega \Delta t\right)}{\lambda_{2}\sin\left(\omega \Delta t\right)}$$

Then, the value of the maximum at $t_{0}$ can be calculated as

(4) $$V\left(t_{0}\right)=\sqrt{\lambda_{1}^{2}+\lambda_{2}^{2}+2\lambda_{1}\lambda_{2}\cos\left(\omega \Delta t\right)}$$

Make $k=\lambda_{1}/\lambda_{2}$, then

(5) $$V\left(t_{0}\right)=\lambda_{2}\sqrt{k^{2}+2k\cos\left(\omega \Delta t\right)+1}$$

- When Δt∈[T/4, T/2)

In this case, as illustrated in Figure 4b, the distance between two debris particles is not very narrow. In Segment I, let

(6) $$V^{\prime }\left(t\right)=\omega \lambda_{1}\cos(\omega \;t)=0$$

We have

(7) $$t_{1}=\pi /2\omega$$

indicating a maximum of $V\left(t_{1}\right)=\lambda_{1}$ in Segment I. Correspondingly, there is a minimum in Segment III.

In Segment II, let

(8) $$V^{\prime }\left(t\right)=\omega \lambda_{1}\cos(\omega \;t)+\omega \lambda_{2}\cos[\omega \left(t-\Delta t\right)]=0$$

having two extreme values. The time index of the maximum is

(9) $$t_{0}=\frac{\pi }{\omega }-\frac{1}{\omega }\arctan\frac{\lambda_{1}+\lambda_{2}\cos\left(\omega \Delta t\right)}{\lambda_{2}\sin\left(\omega \Delta t\right)}$$

as well as the value of the maximum being

(10) $$V\left(t_{0}\right)=\lambda_{2}\sqrt{k^{2}+2k\cos\left(\omega \Delta t\right)+1}$$

- When Δt∈[T/2, 3T/4)

In this case, as shown in Figure 4c, the extreme values in Segments I and III still exist and the maximum in Segment I is still $V\left(t_{1}\right)=\lambda_{1}$. The maximum in Segment II is at $t_{1}=2\pi /\omega$, and we have

(11) $$V\left(t_{1}\right)=-\lambda_{2}\sin(\omega \Delta t)$$

- When Δt∈[3T/4, T)

In this case, as shown in Figure 4d, there is a maximum $\lambda_{1}$ in Segment I and a maximum $\lambda_{2}$ in Segment III.

According to the analysis above, we can conclude that the aliasing affects the shape of the debris signal, especially Debris B’s peak. To help analyze the effect of aliasing, we focused on analyzing three of the waveforms’ features: the value of each detected maximum, number of detected maximums and sum value of the overall detected maximums; correspondingly, these features can indicate the size, number and cumulative weight of the debris particles. Furthermore, to help evaluate the severity of the aliasing, the relative size (*RS*) is proposed to replace size. Specifically,

(12) $$RS=\frac{\lambda_{A}}{\lambda_{N}}$$

where $\lambda_{N}$ is the normal value of the signal’s maximum without aliasing; $\lambda_{A}$ is the value of the maximum after aliasing. Furthermore, the *RS* of the overall size can be defined as $RS_{O}$. As in the case of Equation (5), $V\left(t_{0}\right)$ is the result after aliasing; it represents the sum value of overall maximum. Then, $RS_{O}$ is

(13) $$RS_{O}=\frac{V\left(t_{0}\right)}{\lambda_{1}+\lambda_{2}}=\sqrt{1+\frac{2\cos\left(\omega \Delta t\right)-2}{\frac{\lambda_{1}}{\lambda_{2}}+\frac{\lambda_{2}}{\lambda_{1}}+2}},\;\Delta t\in [0,\;T/4)$$

When the sizes of the debris particles approach each other in a certain stage of wearing, we assume the two debris particles are of similar size in the aliasing, that is, k=1. Then, Equation (13) can be simplified as

(14) $$RS_{O}=\sqrt{1+\frac{\cos\left(\omega \Delta t\right)-1}{2}},\;\Delta t\in [0,\;T/4)$$

Expanding, we have

(15) $$RS_{O}=\{\begin{array}{c} \sqrt{1+\frac{\cos\left(\omega \Delta t\right)-1}{2}},\;\Delta t\in [0,\;T/4)\\ \frac{1+\sqrt{2+2\cos\left(\omega \Delta t\right)}}{2},\;\Delta t\in [T/4,\;T/2)\\ \frac{1-\sin\left(\omega \Delta t\right)}{2},\;\Delta t\in [T/2,\;3T/4)\;\;\\ 1,\;\Delta t\in [3T/4,\;T) \end{array}$$

Similarly, we define the *RS* of Debris B as

(16) $$RS_{B}=\{\begin{array}{c} \sqrt{2\cos\left(\omega \Delta t\right)+2},\;\Delta t\in [T/4,\;T/2)\\ -\sin\left(\omega \Delta t\right),\;\Delta t\in [T/2,3T/4)\\ 1,\;\;\Delta t\in [3T/4,\;T) \end{array}$$

Based on the analysis above, a further summary can be made. In Table 1, three selected features of the aliasing signal are used to present the performance of the two similar debris particles’ aliasing signals when Δt is moving at different intervals. The symbol ↓ means the value of *RS* decreases with an increase in Δt’s value, while the symbol ↑ means the value of *RS* increases with an increase in Δt’s value.

From Table 1, when Δt belongs to the interval of [0, T/4), only one maximum can be detected; the overall size is available but affected more severely with an increase in Δt. In this case, it is impossible to distinguish whether the signal is from a large size debris particle or two small-size debris particles. When Δt is moving from [T/4,T/2) to [T/2, 3T/4), the information of Debris A is unaffected; the information of Debris B becomes worse when Δt increases in the interval of [T/4, T/2), reaching the worst situation in which Debris B’s information vanishes when Δt=T/2, and then becomes better with an increase in Δt in the interval of (*T*/2, 3*T*/4). When Δt moves to the next interval, the aliasing phenomenon disappears. It is obvious that the aliasing is more serious when Δt is in the interval around T/2 than in other intervals. The second severe area is in the interval of [0, T/4).

## 3. Cross-Correlation Algorithm-Based Optimization

A cross-correlation (CC) algorithm means the infinite integrals of two functions that are the complex conjugate and inverse translation are multiplied, or the infinite integration of the first function is the complex conjugate, which is translated in turn and then multiplied by a second function. The calculation formula for cross-correlation is Equations (16) or (17):

(17) $$R_{\mathrm{fh}}\left(x\right)=\int_{-\infty }^{\infty }f^{*}\left(t\right)\;h\left(t+\tau \right)dt$$

(18) $$R_{\mathrm{fh}}\left(x\right)=\int_{-\infty }^{\infty }f^{*}\left(t-\tau \right)\;h\left(t\right)dt$$

Physically, the result of cross-correlation reflects the measure of similarity between two signals. Based on this and the aliasing signal model built in Section 2, the potential of a sliding correlation in dealing with two similar debris particles’ aliasing problem is studied in this section.

### 3.1. Cross-Correlation Analysis of Aliasing Signal

**Figure 5:**
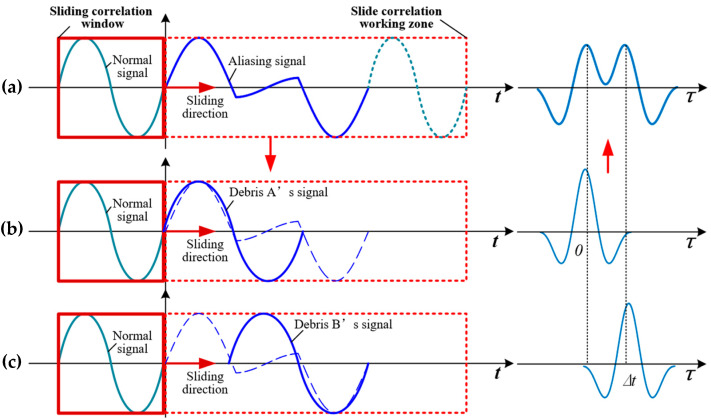
Cross-correlation algorithm; (**a**) the process of cross-correlation (*CC)* for a two-debris aliasing signal (left) and its result (right); (**b**) the process of *CC* for Debris A’s signal and its result; (**c**) the process of *CC* for Debris B’s signal and its result.

In our algorithm, as illustrated in Figure 5, there is a sliding window moving from the beginning of the aliasing signal to the end. In the sliding window, the integration of two functions is operated by each moving step. The result in a whole term can be expressed as follows

(19) $$c\left(\tau \right)=\int_{-\infty }^{\infty }x\left(t-\tau \right)\cdot V\left(t\right)dt$$

where c(τ) is the result of the algorithm, τ is the moving distance of the sliding window, and x(t−τ) represents the waveform of a single debris particle’s normal signal like in Figure 2a. According to Figure 3, where V(t) is the linear addition of two single signals, the process in Figure 5a can be separated into b and c; the result of a is then the linear addition of the results from b and c as illustrated in the right side of Figure 5.

The process in Figure 5b can be expressed as

(20) $$c_{A}\left(\tau \right)=\int_{-\infty }^{\infty }x_{A}\left(t\right)\cdot x\left(t-\tau \right)dt$$

where $x_{A}\left(t\right)$ represents the waveform of Debris A. Equation (19) has a different expression when τ belongs to different intervals.

- When τ∈[−2π/w,0)
  (21) $$c_{A}\left(\tau \right)=\int_{0}^{\tau +2\pi /\omega }\lambda_{1}\sin\left(\omega t\right)\cdot \sin\omega \left(t-\tau \right)dt$$
- When τ∈[0,2π/w)
  (22) $$c_{A}\left(\tau \right)=\int_{\tau +2\pi /\omega }^{2\pi /\omega }\lambda_{1}\sin\left(\omega t\right)\cdot \sin\omega \left(t-\tau \right)dt$$

According to the result of Equations (21) and (22), we have

(23) $$c_{A}\left(\tau \right)=\{\begin{array}{l} 0,\;\tau <-\frac{2\pi }{\omega }\;\mathrm{or}\;\tau >\frac{2\pi }{\omega }\\ \frac{1}{2}\lambda_{1}\left(\tau +\frac{2\pi }{w}\right)\cos\left(\omega \tau \right)-\frac{\lambda_{1}}{2w}\sin\left(\omega \tau \right),\;\tau \in \left[-\frac{2\pi }{\omega },0\right)\\ \frac{1}{2}\lambda_{1}\left(-\tau +\frac{2\pi }{w}\right)\cos\left(\omega \tau \right)+\frac{\lambda_{1}}{2w}\sin\left(\omega \tau \right),\;\tau \in \left[0,\frac{2\pi }{\omega }\right) \end{array}$$

Similarly, the process in Figure 5c can be expressed as

(24) $$c_{B}\left(\tau \right)=\int_{-\infty }^{\infty }\frac{1}{k}\cdot x_{A}\left(t-\Delta t\right)\cdot x\left(t-\tau \right)dt$$

A further transformation of Equation (24) can produce

(25) $$c_{B}\left(\tau \right)=\frac{1}{k}\int_{-\infty }^{\infty }x\left(t_{1}\right)\cdot x\left(t_{1}+\Delta t-\tau \right)dt_{1}=\frac{1}{k}c_{A}\left(\tau -\Delta t\right)$$

where $t_{1}=t-\Delta t$. Then, the process in Figure 5a can be expressed as

(26) $$c\left(\tau \right)=c_{A}\left(\tau \right)+c_{B}\left(\tau \right)=c_{A}\left(\tau \right)+\frac{1}{k}c_{A}\left(\tau -\Delta t\right)$$

According to the analysis above, we can obtain the function of c(τ) as follows:

(27) c(τ)={0,    τ<−2πω  or  τ≥2πω+Δtc1(τ),    τ∈[−2πω,−2πω+Δt)c1(τ)+1kc1(τ−Δt), τ∈[−2πω+Δt,0)c2(τ)+1kc1(τ−Δt),   τ∈[0,Δt)c2(τ)+1kc2(τ−Δt),  τ∈[Δt,2πω)1kc2(τ−Δt),  τ∈[2πω,2πω+Δt)      

where $c_{1}\left(\tau \right)=\frac{1}{2}\lambda_{1}\left(\tau +\frac{2\pi }{w}\right)\cos\left(\omega \tau \right)-\frac{\lambda_{1}}{2w}\sin\left(\omega \tau \right)$, $c_{2}\left(\tau \right)=\frac{1}{2}\lambda_{1}\left(-\tau +\frac{2\pi }{w}\right)\cos\left(\omega \tau \right)+\frac{\lambda_{1}}{2w}\sin\left(\omega \tau \right)$ and 0 ≤Δt≤2π/w.

**Figure 6:**
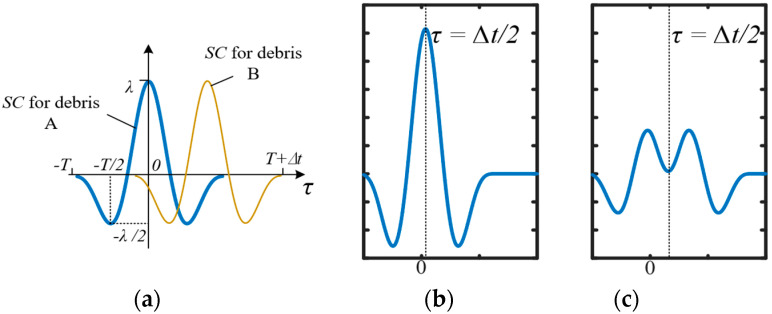
Performance of the *CC* for aliasing signal when Δt moves; (**a**) result of *CC* for Debris A and B’s signal before summing; (**b**) c(τ)’s waveform, Situation 1; (**c**) c(τ)’s waveform, Situation 2.

Assuming the two debris particles are of a similar size, which is k=1, Equation (27) can be simplified. Under this situation, the waveform’s shape is symmetrical, respecting τ=Δt/2. With the moving of Δt, there are two kinds of c(τ) waveform as depicted in Figure 6b,c. With an increase in Δt, c(τ)’s shape will turn from (b) to (c).

Both situations have a peak when τ=Δt/2, while only Situation c presents two maximums. While the peak in Figure 6c is a minimum, it must satisfy the following function

(28) $$\{\begin{array}{c} c^{\prime }\left(\tau \right)=0\\ c^{\prime \prime }\left(\tau \right)>0 \end{array}$$

After dealing with the above function, we have a satisfactory answer for Δt>9π/10w.

- When 0<Δt<9π/10w

As shown in Figure 6b, there is only one maximum at τ=Δt/2, and its value is

(29) $$c\left(\frac{1}{2}\Delta t\right)=\lambda_{1}\left(\frac{2\pi }{\omega }-\frac{1}{2}\Delta t\right)\cos\left(\frac{1}{2}\omega \Delta t\right)+\frac{1}{w}\lambda_{1}\sin\left(\frac{1}{2}\omega \Delta t\right)$$

- When Δt>9π/10w

The peak at τ=Δt/2 is a minimum, as shown in Figure 6c; the maximums are at around τ=0 and τ=Δt:

(30) $$c\left(0\right)=c\left(\Delta t\right)=\frac{\pi \lambda_{1}}{\omega }+\frac{1}{2}\lambda_{1}\left(\frac{2\pi }{\omega }-\Delta t\right)\cos\omega \Delta t+\frac{\lambda_{1}}{2\omega }\sin\omega \Delta t$$

Similarly to in Section 2, we select the characteristics of the number of detected maximums, the relative size of Debris B’s *CC* maximum $R{S_{B}}^{\prime }$, and the relative size of the overall maximums. While the *CC* result of one debris signal along is $\frac{\lambda_{1}\pi }{w}$, we have

(31) $$RS_{O}^{\prime }=\{\begin{array}{c} \frac{c\left(\Delta t/2\right)}{2\lambda_{1}\pi /\omega }=\left(1-\frac{\omega }{4\pi }\Delta t\right)\cos\left(\frac{1}{2}\omega \Delta t\right)+\frac{1}{2\pi }\sin\left(\frac{1}{2}\omega \Delta t\right),\;0<\Delta t<9\pi /10w\\ \frac{c\left(0\right)+c\left(\Delta t\right)}{2\lambda_{1}\pi /\omega }=1+\left(1-\frac{\omega \Delta t}{2\pi }\right)\cos\omega \Delta t+\frac{\sin\omega \Delta t}{2\pi },\;\Delta t>9\pi /10w \end{array}$$

and

(32) $$R{S_{B}}^{\prime }=\frac{c\left(\Delta t/2\right)}{\pi \lambda_{1}/\omega }=1+\left(1-\frac{\omega \Delta t}{2\pi }\right)\cos\omega \Delta t+\frac{\sin\omega \Delta t}{2\pi },\;\Delta t>9\pi /10w$$

Similarly to in Section 2, a table is made to sum up the performance of the aliasing signal’s three selected characteristics after a *CC*. To sum up, a table can be obtained such as Table 2.

### 3.2. Optimization Strategy for Aliasing Signal Processing

From the above analysis, we have the function of *RS*_O_ and $RS_{O}^{\prime }$, $RS_{B}$ and $R{S_{B}}^{\prime }$. As the value of these functions reflects the aliasing signal’s information, a comparation between them can help to determine the most optimized strategy for evaluating the affected debris’ peak value.

**Figure 7:**
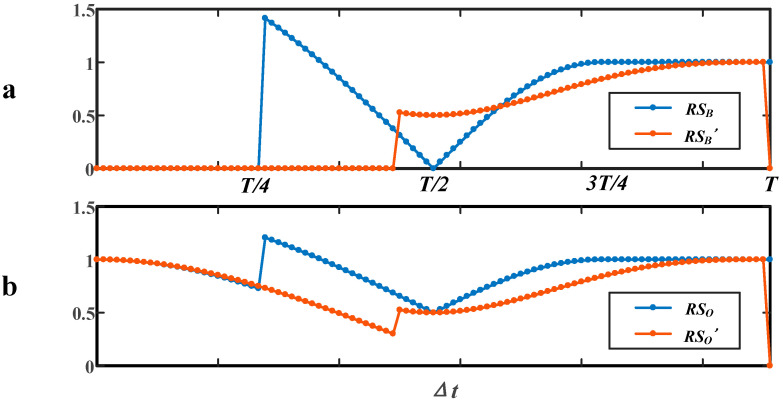
Comparation of relative size (*RS*) before and after a *CC.* (**a**) Debris B; (**b**) Overall.

As depicted in Figure 7, the values of the different *RS* change successively with the moving of Δt from 0 to *T*; the more the values of *RS* approach 0, the worse the situation; contrarily, the more the values of *RS* approach 1, the better the situation. Here, we can find again the severest cases figured out in Section 2, which are when Δt is in the interval around T/2 and interval of [0, T/4). From Figure 7a, the *RS* after *CC* ($R{S_{B}}^{\prime }$) around T/2 is above the *RS* without processing ($RS_{B}$), apparently. However, the values of $R{S_{B}}^{\prime }$ at other intervals are not closer to 1 than the values of $RS_{B}$. From Figure 7b, the overall *RS* after *CC* ($RS_{O}^{\prime }$) at the interval of [0, T/4) is a little above the overall *RS* without processing ($RS_{O}$). Similarly, the values of $RS_{O}^{\prime }$ at other intervals are not closer to 1 than the values of $RS_{O}$. The result of the comparison proves the advantage of *CC* in optimizing the severe cases of aliasing.

In order to make a comparison easier, the interval of Δt can be divided into four segments. In Segment I, Δt belongs to [0, T/4); in Segment III, Δt is around the interval of T/2; Segment II is between Segment I and Segment III; Segment IV is after Segment III and ends in *T*. In Table 3, a summary of the comparison is given. We use “OS” to represent the original aliasing signal without processing and “SC” to represent the signal after *CC*. In Segment III, while the *CC* can help to detect Debris B, the maximum representing Debris A is also affected, thus making $RS_{O}^{\prime }$ < $RS_{O}$; the preferred strategy is combining Debris A’s signal from OS and Debris B’s information from SC.

**Figure 8:**
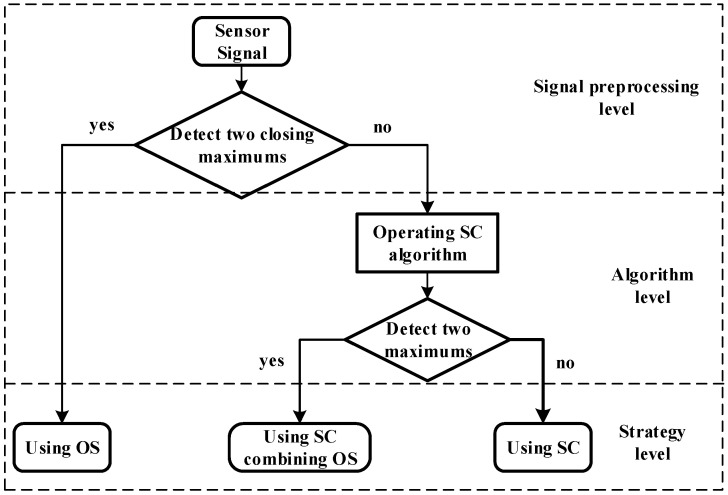
Aliasing signal processing strategy.

Based on the study above, we can adopt the following strategy as shown in Figure 8.

When the sensor has detected two closing maximums (within one cycle *T*), the original signal is used. When there is only one maximum detected (within one cycle *T*), the *CC* will be operated. If there are two maximums detected after *CC*, the value of Debris B’s maximum $\lambda_{2}$ can be evaluated by

(33) $$\bar{\lambda_{2}}=\lambda_{1}\cdot \frac{c_{2}}{c_{1}}$$

where $\lambda_{1}$ is the value of the maximum detected from the original signal, $c_{1}$ is the first value of the detected SC’s maximums and $c_{2}$ is the second value of the detected SC’s maximums.

## 4. Experiment Validation

### 4.1. Simulation Experiment

This section describes a simulated experiment that was carried out first. As in a practical system, the debris signal is accompanied by various kinds of interferences [22]; we built the original signal by combining an aliasing signal as described in Section 2, an interference of specific frequency and random noise. The specific parameters are provided in Table 4.

**Figure 9:**
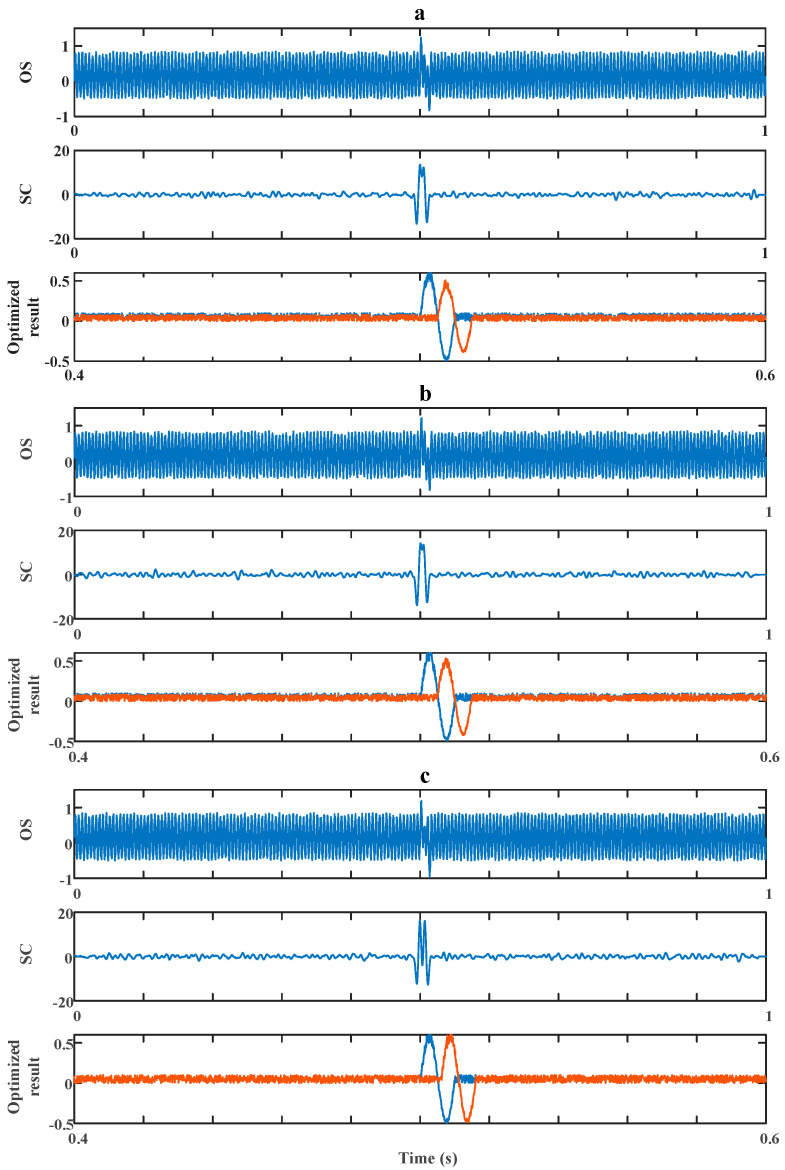
Method’s performance for signals of low signal-to-noise ratio (*SNR*) while *k* = 1. (**a**) Δt=0.5T. (**b**) Δt=0.48T. (**c**) Δt=0.6T.

**Figure 10:**
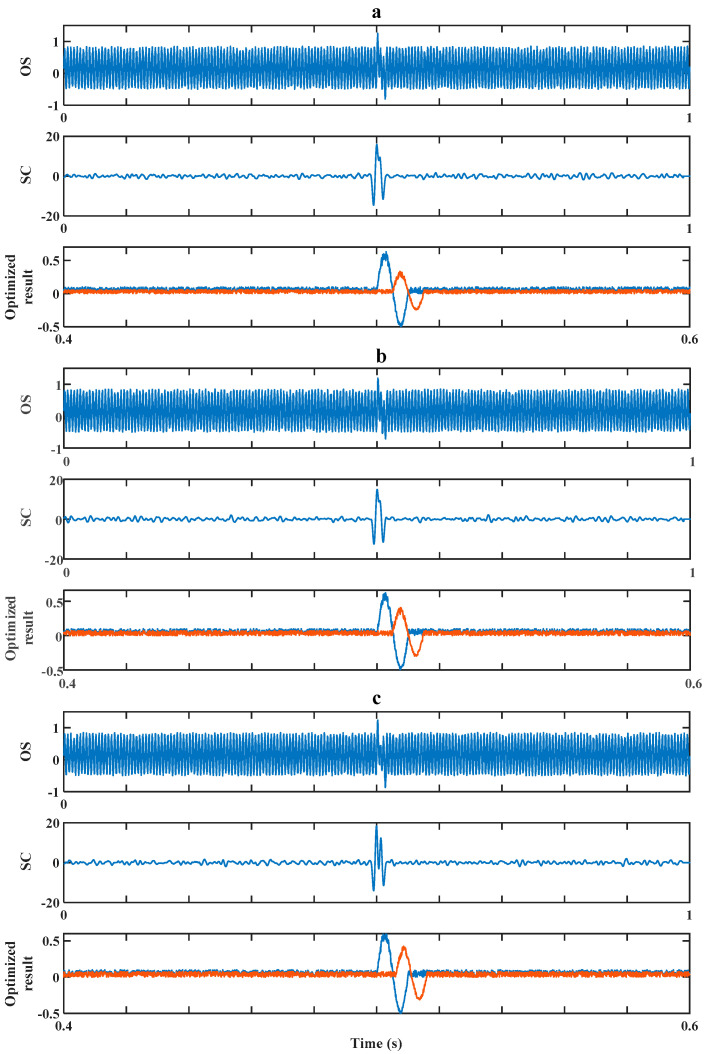
Method’s performance for signals of low *SNR* while *k* = 1.2. (**a**) Δt=0.5T. (**b**) Δt=0.48T. (**c**) Δt=0.6T.

**Figure 11:**
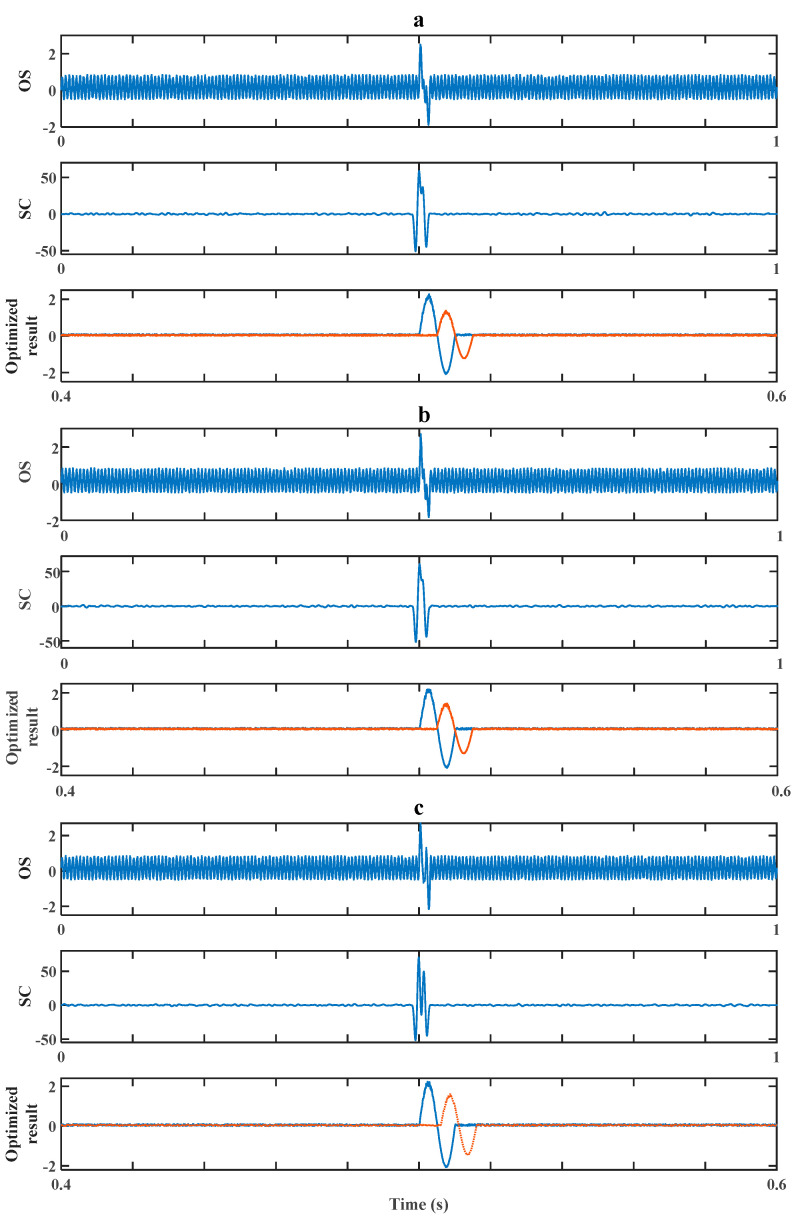
Method’s performance for signals of high *SNR* while *k* = 1.2. (**a**) Δt=0.5T. (**b**) Δt=0.48T. (**c**) Δt=0.6T.

The efficiency of our method in dealing with the worst situation (in which Δt is in the interval around T/2) was then verified by a simulation experiment. There are three sets of experiments corresponding to Figure 9, Figure 10 and Figure 11. In each figure, there are three pictures, which are Picture (a), Picture (b) and Picture (c), representing three different values of Δt around T/2, which are 0.5T, 0.48T and 0.6T, correspondingly. In Pictures (a), (b) and (c), the OS of each represents the original signal without processing; the SC of each represents the result after a cross-correlation algorithm; the Optimization Result of each shows both debris signals’ evaluation results after the optimization strategy.

Firstly, we experimented on cases of signals with low signal-to-noise ratios (*SNRs*; the signals’ amplitudes were set to be 0.5) as shown in Figure 9 and Figure 10. In Figure 9, we consider the situation of k=1 ($k=\lambda_{1}/\lambda_{2}$, which is the ratio between Debris A’s and Debris B’s amplitude), which indicates two aliasing debris particles of the same size. In Figure 10, we consider the situation of k=1, which indicates two aliasing debris particles of different sizes.

In Figure 9a–c, only one maximum was detected and unaffected from OS, which means the information of the second debris particle was totally lost. After *CC* processing, two maximums were detected in SC. Then, after the optimization strategy, both debris particles’ sizes could be evaluated and shown in the optimization results. Only by OS, it is obvious that no sign of other debris can be found. By using the above method, the missing debris was found, and its amplitudes were evaluated to be 0.4, 0.43 and 0.49, corresponding to Figure 9a–c, which are 80%, 86% and 98% (calling this value the evaluating accuracy, which ranges from 0% to 100%; the larger, the more accurate) of their real amplitudes (which are 0.5). Correspondingly, the evaluation of the overall sizes (which are the sums of two debris particles’ amplitudes) accounts for 90%, 97% and 99% of the overall evaluating accuracy. The result indicates that the distance from the most severe aliasing situation (Δt=T/2) has a positive effect on the evaluating accuracy.

Then, making k=1.2 in the following simulation, Δt had the same values as above, which were 0.5T, 0.48T and 0.6T. The result is shown in Figure 10. Similarly, the second debris was detected and its size was evaluated with an evaluating accuracy of 60%, 73.5% and 77% as well as overall evaluating accuracy of 80%, 86.7% and 88.5%. Compared to when k=1, the evaluation result was less accurate when k=1.2, which indicates the difference between the aliasing debris particles’ sizes has a negative effect on the evaluation results.

Next, we experimented on cases of signals with high signal-to-noise ratios (the signals’ amplitudes were set to be 2). Making k=1.2 in the following simulation, the simulation result when Δt was around T/2 is shown in Figure 11. Similarly, the second debris was detected and its size was evaluated with an evaluating accuracy of 70.6%, 74.5% and 83.3% as well as evaluating accuracy of 85.3%, 87.3% and 91.6%. Compared to Figure 10, there was an improvement in the evaluation’s accuracy. The result indicates noise has a negative effect on the evaluation result. Figure 10 and Figure 11 also indicate again the distance from the most severe aliasing situation (Δt=T/2) has a positive effect on the evaluating accuracy.

### 4.2. Wax Block Experiment

**Figure 12:**
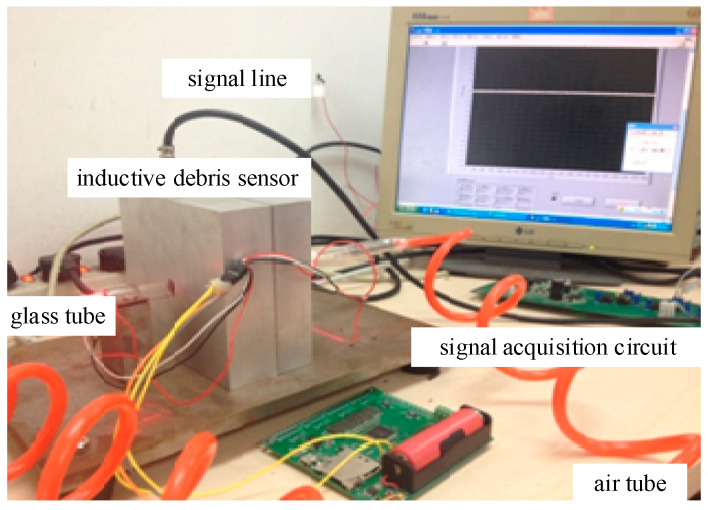
Real wax block experimental system.

From the above simulation analysis, we have the aliasing issue effect existing in the conditions of both high and low *SNR*; the effectiveness of our method was verified; our method also has high reliability in a noisy condition. Next, we discuss a real system as shown in Figure 12; the experimental system generally included an inductive debris sensor designed by Hong, a signal acquisition circuit, a signal processing system and the corresponding components. The main parameters were decided as shown in Table 5.

**Figure 13:**
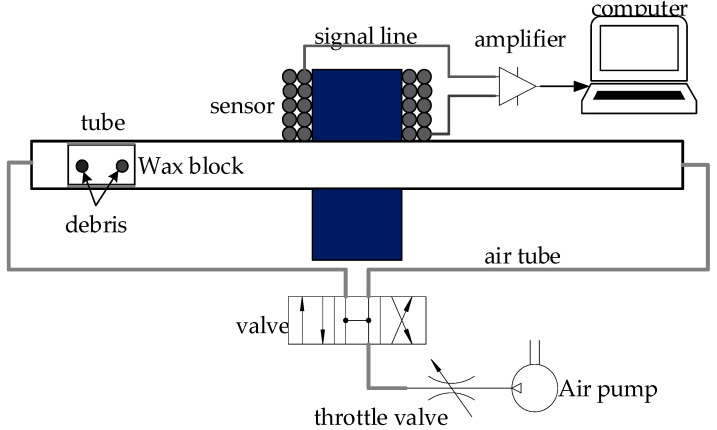
Wax block experimental system schematic.

Meanwhile, in the real experiment, many factors were uncontrollable. In order to create a favorable environment, many factors were considered. For example, the air pump was used to create an air flow of fixed velocity in the air tube; the wax block was used to fix the location of the debris particles so that the distance between the two signals was controllable; the single debris’ signal was acquired with a signal acquisition from single debris; to avoid the interface of other noise, debris of a large size was used in the experiment; the amplifier magnification was chosen to be 900 times (maximum, 4000) so as to increase the amplitude of the debris signal while avoiding excessive power frequency interference. The system schematic of the experiment is shown in Figure 13.

**Figure 14:**
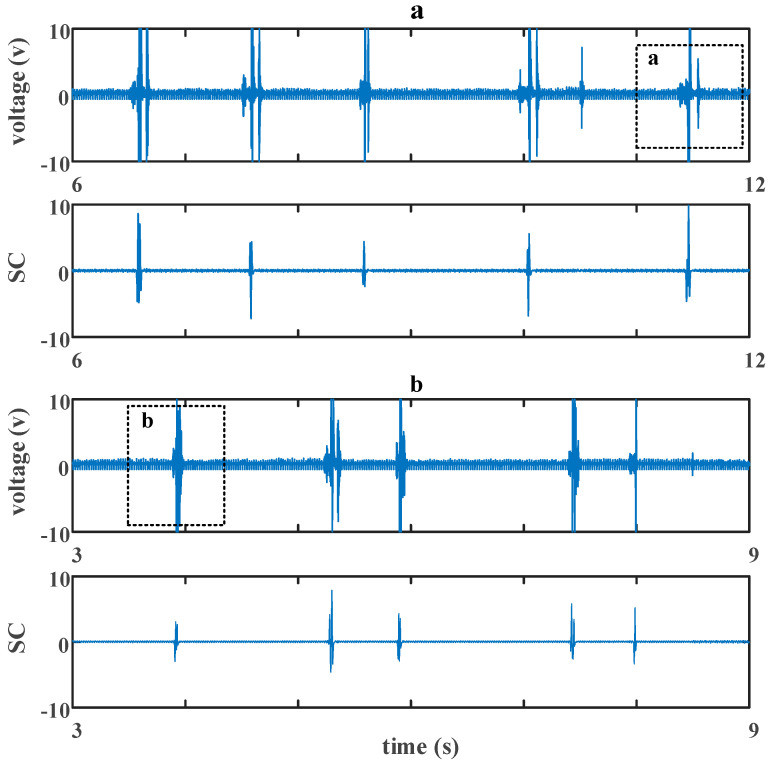
Performance of an experimental term when the space was 3 cm (**a**) and 6 cm (**b**).

**Figure 15:**
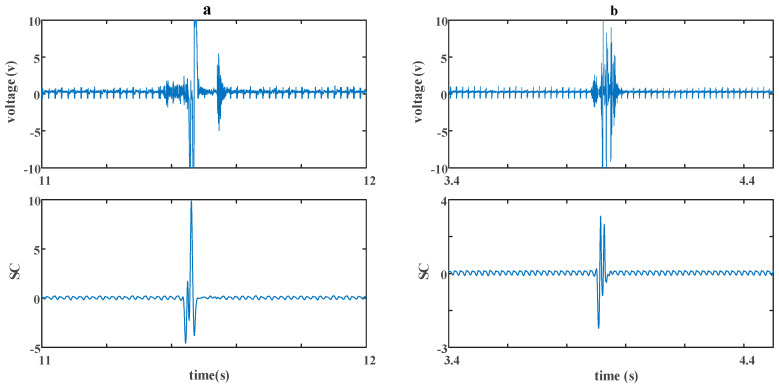
A detailed view of the dashed boxes in Figure 14; (**a**) a detailed view of dashed box a; (**b**) a detailed view of dashed box b.

The experimental result is shown in Figure 14. In both Figure 14a,b, the first picture of each is the original signal, and the second picture of each is the result after a *CC.* In Figure 14a, the space between two debris wax blocks was set to be 3 cm, while it was 6 cm in Figure 14b. A detailed view of the dashed boxes a and b is shown in Figure 15.

In Figure 15a, the original signal is mixed with two debris particles’ signals, a wax block’s signal and some other interference. Besides the interference’s signal, there is only one maximum detected, which means a typical aliasing problem as mentioned earlier. After a *CC*, two maximums are detected, which indicates the second debris, while at the same time, the interference’s signal and wax block’s signal are also decreased; that is because similarity between the debris’ signal and these signals is very rare, so a peak will not present when a *CC* is operating through them. In Figure 15b, there are several maximums detected from the original signal, which is not a traditional situation. This may result from an untraditional aliasing or other interferences, while the result from *CC* is satisfied.

## 5. Conclusions

This paper mainly focuses on two parts of work. Firstly, we used a sinewave-based mathematical model to analyze a debris signal’s aliasing behavior. The value of the aliasing model’s maximum was studied in detail, and three corresponding features, which are the number, size and cumulative size of the debris particles, were used to evaluate the severity of the aliasing problem. On this basis, the most severe situation was figured out based on two similar debris particles’ aliasing analysis. Next, the potential of using a cross-correlation algorithm in dealing with aliasing’s severest situation was studied. While operating a cross-correlation algorithm, the debris’ signal was emphasized in its progress. The emphasizing will come out as a maximum every time it encounters a debris signal, which is a solution to the debris aliasing signal’s severest situation. After this, a comparison between the original signal and a cross-correlation-processed signal was made, on which an optimization strategy was based. The simulation and experimental results also verified the efficiency of our method. In a following study, we will consider expanding our method to more complex conditions, such as the aliasing of more than two debris particles, and then experiment in a more practical system, such as a mechanical system with an oil pipe.

## Figures and Tables

**Table 1:** Performance of two similar debris particles’ aliasing.

Δt	Number of Detected Debris	*RS*_O_ (Overall Size)	*RS_B_* (Debris B)
[0, T/4)	1	↓	——
[T/4, T/2)	2	↓	↓
T/2	1	1/2	0
(*T*/2, 3*T*/4)	2	↑	↑
[3T/4, T)	2	1	1

**Table 2:** Performance of two similar debris particles’ aliasing after *CC*.

Δt	Number of Detected Debris	$RS_{O}^{\prime }\;(\mathrm{Overall}\;\mathrm{Size})$	$R{S_{B}}^{\prime }\;(\mathrm{Debris}\;B)$
[0, 9T/20)	1	↓	——
[9T/20, T)	2	↑	↑

**Table 3:** Detailed comparison between the original aliasing signal (OS) and signal after CC (SC).

Δt	Number of Detected Debris Particles		*RS* of Overall Size		*RS* of Debris B		Preferred
	OS	SC	$RS_{O}$	$RS_{O}^{\prime }$	$RS_{B}$	$R{S_{B}}^{\prime }$	
I	1	1	$RS_{O}^{\prime }$ > $RS_{O}$		-	-	SC
II	2	1	$RS_{O}^{\prime }$ < $RS_{O}$		>50%	-	OS
III	1	2	$RS_{O}^{\prime }$ < $RS_{O}$		-	>50%	SC and OS
IV	2	2	$RS_{O}^{\prime }$ < $RS_{O}$		1	<1	OS

**Table 4:** Simulation parameters.

Parameter	Value
Frequency of debris signal *w*	100 Hz
Amplitude of inference	0.5
Amplifier of noise	0.2
Length of correlation *T*	0.01 s
Sampling frequency	10 kHz

**Table 5:** Parameters of the experimental system.

Parameter	Value
Velocity of debris particle	5 m/s
Size of particle	200 μm
Amplifier magnification	900 times
Space between two debris particles	3 cm/6 cm
Sampling frequency	10 kHz
